# Eyesi direct ophthalmoscope simulator: an effective training tool for medical undergraduates

**DOI:** 10.1186/s12909-024-05780-w

**Published:** 2024-07-20

**Authors:** Canying Liu, Jicheng Lin, Siting Wu, Yingting Zhu, Yuxian Zou, Qi Zhang, Zhidong Li, Yehong Zhuo, Yiqing Li

**Affiliations:** State Key Laboratory of Ophthalmology, Zhongshan Ophthalmic Center, Guangdong Provincial Key Laboratory of Ophthalmology and Visual Science, Sun Yat-sen University, Guangzhou, 510060 China

**Keywords:** Direct ophthalmoscope, Simulator, Fundus examination, Medical education, Primary medical institutions

## Abstract

**Introduction:**

Non-ophthalmologists often lack sufficient operational training to use a direct ophthalmoscope proficiently, resulting in a global deficit of basic ophthalmological skills among general practitioners. This deficiency hampers the timely diagnosis, referral, and intervention of patients. Consequently, the optimization of teaching tools and methods to enhance teaching efficiency is imperative. This study explores the effectiveness of the Eyesi Direct Ophthalmoscope Simulator (Eyesi) as an innovative tool for fundus examination training.

**Methods:**

Medical undergraduates were randomly assigned to Group A or B (*n* = 168). All participants completed a pre-training questionnaire. Group A received Eyesi training, while Group B underwent traditional direct ophthalmoscope (TDO) training. Subsequently, participants answered questionnaires relevant to their respective training methods. Both groups exchanged training tools and completed a summary questionnaire.

**Results:**

After training, 54.17% of participants believed that images presented by the Eyesi were consistent with the real fundus. Group A scored significantly higher than Group B in fundus structure recognition and self-confidence in examination. The degree of mastery over fundus theory score increased from 6.10 ± 0.13 to 7.74 ± 0.16 (*P* < 0.001) in Group A, but Group B did not demonstrate a significant difference. We also compared undergraduates’ tendencies for different learning purposes, 75.59% of participants preferred the Eyesi to TDO as a training tool, and 88.41% of participants were receptive to introducing the Eyesi in training.

**Conclusion:**

According to subjective participant feedback, Eyesi outperformed TDO in fundus observation, operational practice, and theoretical learning. It effectively equips undergraduates with fundus examination skills, potentially promoting the use of direct ophthalmoscopes in primary medical institutions.

**Supplementary Information:**

The online version contains supplementary material available at 10.1186/s12909-024-05780-w.

## Introduction

The transparency of the tissues in front of the retina, along with retina itself, enables unhindered light transmission, facilitating direct visualization of the retina’s superficial vascular and neural structures. Direct ophthalmoscopes are portable devices for the observation of the fundus and the assessment of retinal diseases. A timely and precise direct ophthalmoscopy examination can not only safeguard a patients’ eyesight, such as instances of retinopathy of prematurity, retinal vascular obstruction and retinal detachment, but also their life in critical situations such as uveal melanomas, elevated intracranial pressure, malignant hypertension, and meningitis [1–4]. Direct ophthalmoscopy examination also plays an important role in the early diagnosis, follow-up, and efficacy evaluation of systemic and common eye diseases (i.e. diabetes, hypertension, atherosclerosis, and glaucoma) [5–11]. Although studies have shown that direct ophthalmoscopy is less sensitive than fundus photography for screening eye diseases like diabetic retinopathy, this tool offers the advantages of of great availability, low operational costs, short examination times, and high specificity in detecting sight-threatening eye diseases [12]. For general practitioners and non-ophthalmologists, the direct ophthalmoscope is the preferred equipment for fundus examination and is particularly suitable for primary medical services. Therefore, the ability to utilize the direct ophthalmoscope is a required skill for medical undergraduates and general practitioners in many countries [13–15]. However, the operational training received by most clinical medical undergraduates and general practitioners is typically insufficient for the use of a direct ophthalmoscope, oftentimes leading to inaccurate identification of common fundus lesions. The lack of basic ophthalmological skills among general practitioners has become a global problem, affecting timely diagnosis, referral, and intervention of patients [7, 13, 16–23]. Optimizing the teaching tools and procedures in order to improve teaching efficiency has become a pressing issue that requires an alternative solution.

Problems exist in traditional direct ophthalmoscope (TDO) teaching, including learning difficulties that result in a lack of confidence, difficulty identifying fundus structures and diagnosing retinal diseases, and difficulty properly evaluating students’ fundus examination ability [24–26]. Moreover, TDO training may be hindered by limited access for students to practice on patients, due to the limited number of patients available with each disease at a given time and the reluctance of patients due to discomfort during the examination. In order to address these issues, we introduced the Eyesi Direct Ophthalmoscope simulator (Model EDO491 #03 × 0127, Platform 2.1, Software v1.8.0.113443, VRmagic GMBH, Mannheim, Germany) [27], which simulates the shape and all functions of a real direct ophthalmoscope and reaction of patients such as the changing of pupil size with the lighting level. It provides a built-in case database that allows students to learn independently, and offers a test mode that can be used to evaluate skill mastery. It can provide timely feedback on whether there is retinal diseases in the inspected area from the user’s perspective. Teachers can also provide guidance during the operation through the monitoring screen connected to the simulator, and can check the students’ operation scores in real-time through the VRmNet service [2, 27]. Experienced doctors considered the simulator realistic and found it met the training needs on how to perform direct ophthalmoscopy [28]. Currently, the device is not widely used. The objective of this study was to evaluate whether the Eyesi, as a training tool for fundus examination, can address and rectify issues that currently exist in TDO teaching.

## Methods

This prospective, randomized, controlled trial was conducted at Zhongshan Ophthalmic Center, Guangzhou, Guangdong, China.

### Study participants

Fourth-year medical undergraduates at Sun Yat-Sen University who came to Zhongshan Ophthalmic Center for ophthalmic preclinical training were included in this study. The study received ethics approval via the Ethics Committee at Zhongshan Ophthalmic Center, Sun Yat-sen University and all participants provided written informed consent prior to taking part. All aspects of the study conformed to the tenets of the Declaration of Helsinki. Our inclusion criteria were as follows: (1) successfully completed the theoretical Ophthalmology course; (2) willingness to participate in the entire study. Our exclusion criteria were as follows: (1) refusal to participate; (2) those who had already learned to operate a direct ophthalmoscope prior to this study.

### Questionnaires design

There were four questionnaires administered throughout the duration of the study: the pre-training questionnaire, the questionnaire after the Eyesi training, the questionnaire after TDO training, and the summary questionnaire (Supplementary Tables 1–4). By referring to relevant literatures, considering the difficulties expressed by students in the previous training and consulting clinical teachers and professors, we set up a series of questionnaires. Composed of basic information, views before and after different training, these questionnaires covered the content assessed in previous studies [24, 29, 30] and provided an assessment of teaching effectiveness based on participants’ self-perception. Consisting of single-choice questions, multiple-choice questions, and short answers, they were built according to the first two levels of Kirkpatrick model [31, 32], a four-level evaluation model developed by Donald Kirkpatrick. The first level is about students’ reaction, such as their interest and motivation. The second level is to measure whether students have learned knowledge and/or skills. The questionnaire items were categorized into four dimensions: (1) the importance of learning direct ophthalmoscope operation, (2) competency in direct ophthalmoscope operation, (3) level of theoretical knowledge related to direct ophthalmoscope, and (4) interest in further learning. Questions for each dimension are listed in Supplementary Tables 1–4. And the internal consistency analysis was performed by computing the Cronbach’s alpha and composite reliability statistics (Supplementary Table 5).

### Instructional design

**Fig. 1:**
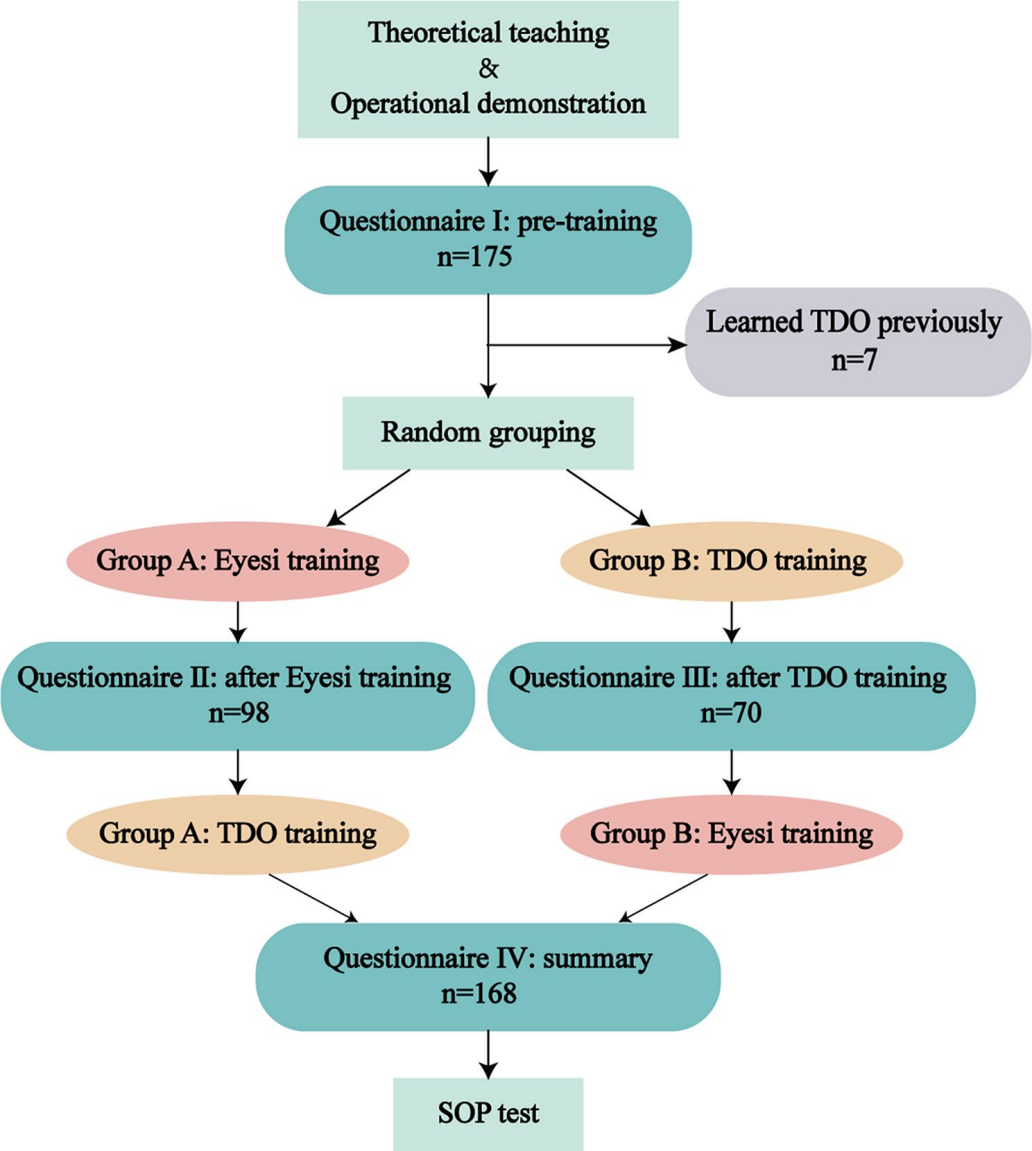
Instructional design of direct ophthalmoscope

A total of 175 undergraduates who came for ophthalmic preclinical training were divided into six groups; approximately 30 students attended ophthalmic preclinical training at each designated teaching slot. Participants received theoretical teaching including the composition of TDO and its operation. And then one instructor demonstrated the operation of TDO and the Eyesi. The instructors and teaching content remained consistent across all students. Subsequently, students were randomly divided into the Eyesi (Group A) or TDO (Group B) group. Participants were required to complete their corresponding questionnaires, and thereafter the two groups exchanged their training tools. Students filled in the summary questionnaire after completing both training sessions and were given the test of standard operation procedure (SOP). The standards of grading are shown in Supplementary Table 6. Instructional design of direct ophthalmoscope is shown in Fig. 1.

**Fig. 2:**
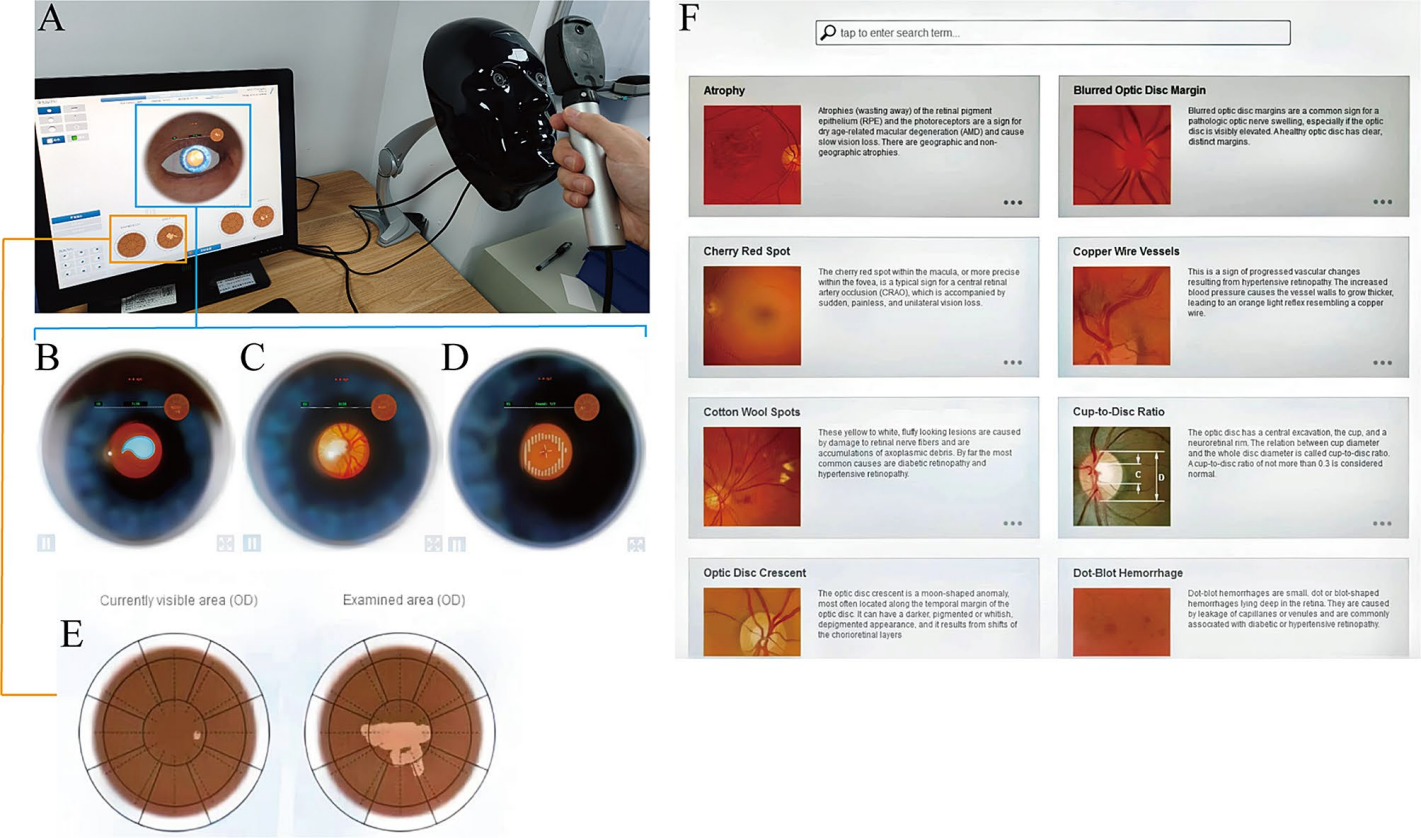
Training Process of the Eyesi Simulator. **A**) The Eyesi simulator consists of three main hardware components: a display monitor, a head-face model, and an ophthalmoscope handle. The screen displays fundus images observed through the ophthalmoscope, showing **B**) blue landmark in droplet shapes, **C**) optic disc structures, and **D**) peripheral retinal hemorrhages. **E**) The screen displays the currently examining area through the ophthalmoscope handle and the already examined area during the fundus examination. **F**) The Eyesi simulator provides an introduction to various typical lesions. (**A**: Image captured by the author; **B**-**F**: Screenshots from the Eyesi simulator display)

For Eyesi training, participants would undergo the following training programs (Fig. 2): (1) Learning the construction and usage of the direct ophthalmoscope handle of the Eyesi simulator; (2) Identifying scattered landmarks on retina (e.g., triangles, crosses) under mydriatic conditions and repeating the process under normal pupillary conditions; (3) Locating retinal landmarks (e.g., retinal arterioles and venules, macula, optic disc) on a simulated normal retina under both mydriatic and normal pupillary conditions and assessing the cup-to-disc ratio while improving the examination coverage by monitoring the examined area (bright zone) and the unexamined area (dark zone) displayed on the monitor; (4) Examining the retina of typical cases under normal pupillary conditions, identifying pathological areas, and making diagnoses of the lesions. Students also learned about the disease progression and retinal lesion descriptions through the simulator’s built-in case library. Throughout the process, the instructing teachers could monitor observed images of students on the monitor and provide timely assistance, such as helping students adjust the position of the ophthalmoscope to ensure a complete examination of the central and peripheral retina and pointing out any overlooked retinal anatomical structures or pathological areas.

For TDO training included the following training programs: (1) Learning the construction and usage of the traditional direct ophthalmoscope; (2) Students observed each other’s fundus structures, and mydriatic eye drops were provided. Students were allowed to decide voluntarily whether to perform mutual mydriatic fundus examinations; (3) Instructors selected suitable and willing patients for examination, allowing students to study the fundus appearances based on patient medical records and direct ophthalmoscope observations.

### Statistical analysis

Data were entered using Microsoft Excel 2017 and all statistical analyses were performed using IBM SPSS Statistics for Windows version 26 (IGM Corp.; Armonk, New York, USA). The Pearson correlation coefficient was used to assess the correlation between the four dimensions of the questionnaires and the training status as well as the training tools. One-way analysis of variation was performed to analyze the difference in the mean scores of undergraduates’ degree of mastery of theoretical knowledge, willingness to use the direct ophthalmoscope in future practice, and interest in further learning, before and after training with different tools. Since the data were not normally distributed, Tamhane’s T2 method was used for post hoc testing. An independent-sample t-test was performed to analyze the difference in the mean scores of fundus structure identification, ease of use, and operation confidence with different training tools. A p-value of < 0.05 was considered statistically significant. The mean (M) ± standard error of mean (SEM) is used to describe the mean of all scores in this paper.

## Results

### Basic information

A total of 168 medical undergraduates were included in this survey, after excluding seven undergraduates with direct ophthalmoscope experience prior to the study. Participants were aged between 20 and 24 years, of whom 51.19% were male. All students had completed the theoretical ophthalmology course (the 9th Edition, published by the people’s Health Publishing House, China) prior to commencement of the study.

### Correlation analysis

The results indicate a significant correlation between the training status (whether training was received or not) and three dimensions: the importance of learning direct ophthalmoscope operation, competency in direct ophthalmoscope operation, and the level of theoretical knowledge related to direct ophthalmoscope (Table 1). Additionally, there was a significant correlation between the type of training tool used and all four dimensions, encompassing interest in further learning as well as the aforementioned three dimensions (Table 2).

**Table 1:** Correlation analysis between training status and the four dimensions of the questionnaires

	①	②	③	④	⑤
➀ **Training**	1				
➁ **Importance**	0.864^***^	1			
➂ **Operation**	0.687^***^	0.755^***^	1		
➃ **Theory**	0.272^***^	0.513^***^	0.539^***^	1	
➄ **Interest**	-0.024	0.304^***^	0.269^***^	0.632^***^	1

***, *P* < 0.001, significant correlation

**Table 2:** Correlation analysis between different training tool and the four dimensions of the questionnaires

	①	②	③	④	⑤
➀ **Tools**	1				
➁ **Importance**	0.315^***^	1			
➂ **Operation**	0.505^***^	0.483^***^	1		
➃ **Theory**	0.392^***^	0.692^***^	0.682^***^	1	
➄ **Interest**	0.346^***^	0.715^***^	0.534^***^	0.725^***^	1

***, *P* < 0.001, significant correlation

### Views before training

#### Direct ophthalmoscopy was considered commonly used and effective clinically but difficult to learn

**Fig. 3:**
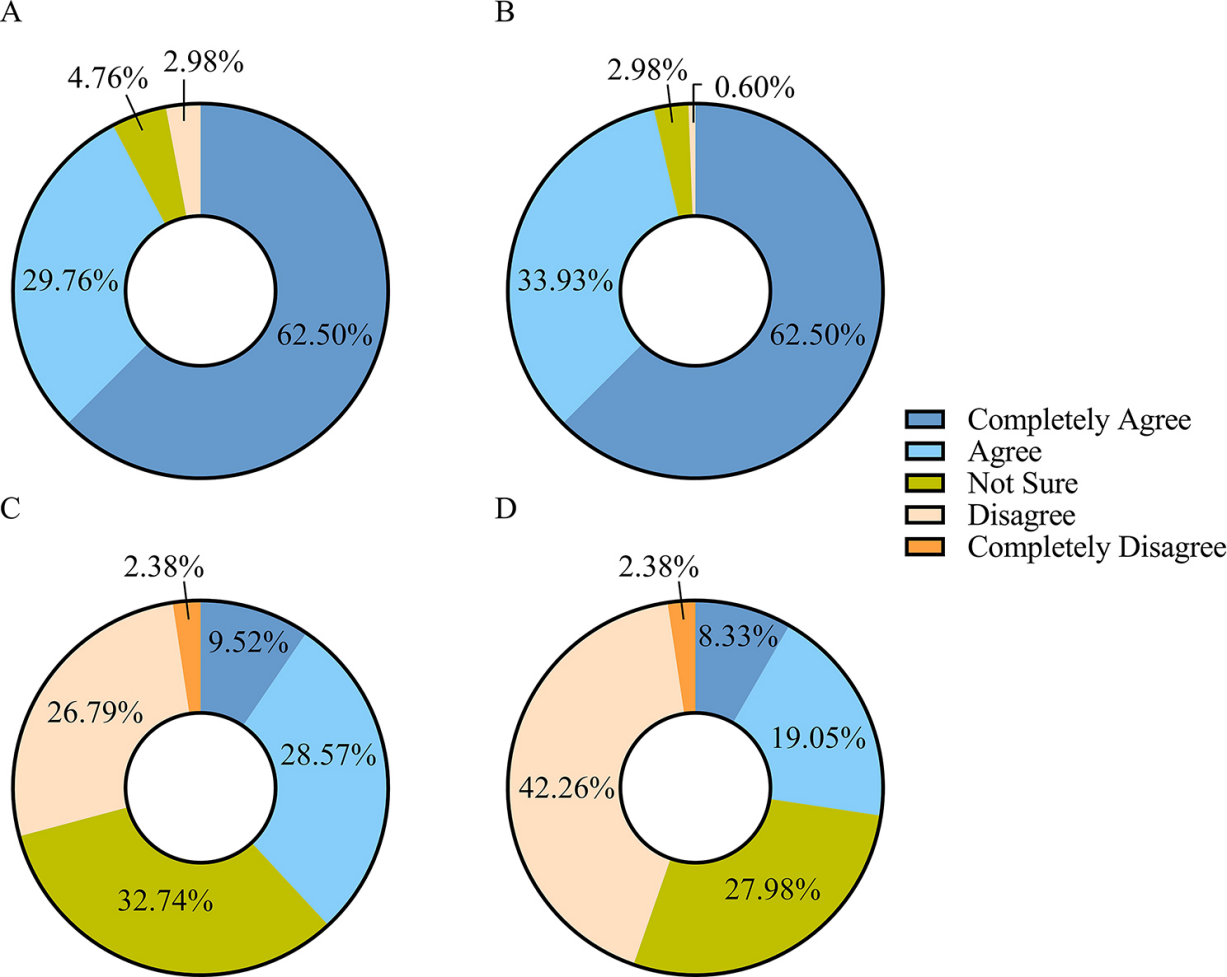
Respondents’ comments on the following statements: **A** Direct ophthalmoscope is one of the most commonly used inspection tools in clinical ophthalmology; **B** Direct ophthalmology is an effective inspection method for the diagnosis of retinal diseases; **C** Learning to use the direct ophthalmoscope is difficult; **D** The examination steps involved in using the direct ophthalmoscope are complex. The questionnaire response rates for the questions represented in this figure is 100%

92% of respondents agreed that the direct ophthalmoscope is one of the most commonly used inspection tools in clinical ophthalmology; 96% agreed that it is an effective inspection method for the diagnosis of retinal diseases (Fig. 3 AB). However, only 29.17% of the respondents believed that the tool was easy to operate while 44.64% believed that the examination was simple (Fig. 3 CD).

#### Direct ophthalmoscopy was considered necessary to learn and attracted the interest of respondents

**Fig. 4:**
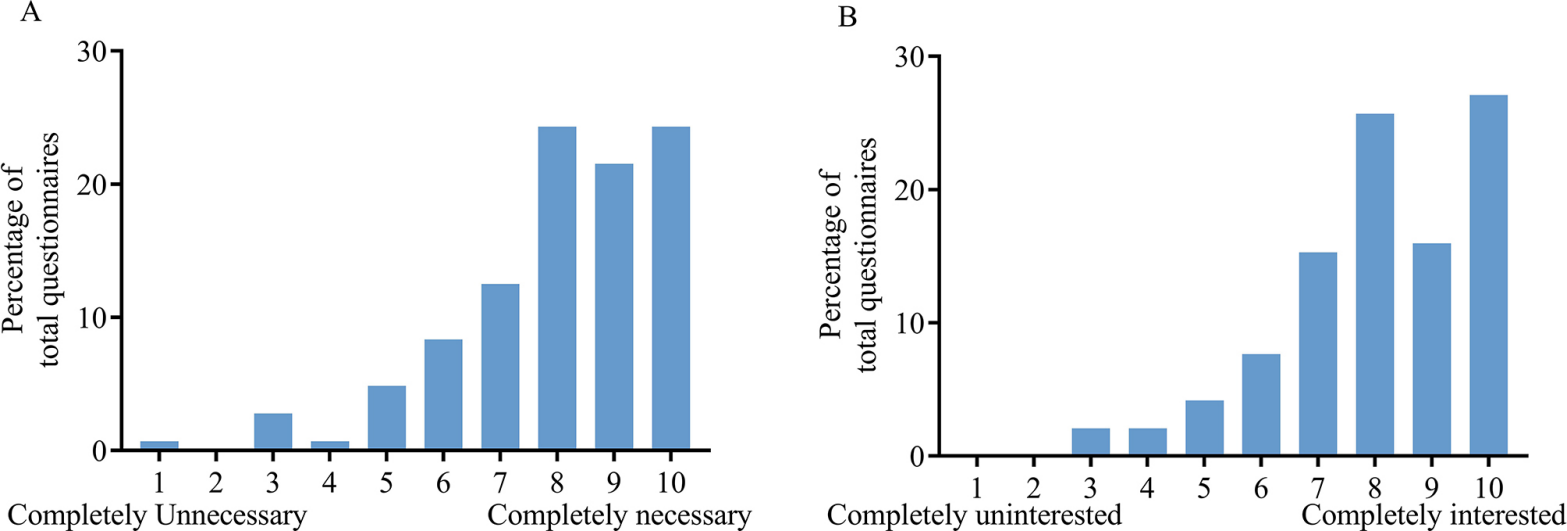
Percentage of each score given by respondents to the following questions: **A** Do you think it is necessary for non-ophthalmologists to master the use of the direct ophthalmoscope? **B** Are you interested in learning to operate the direct ophthalmoscope? The questionnaire response rates for the questions represented in this figure is 85.71%

70% of the respondents rated a score of eight or higher with regard to the necessity for non-ophthalmologists to master operating a direct ophthalmoscope (Fig. 4A). Similarly, 68.74% of the respondents gave a score of eight or higher with regard to their interest in learning to operate a direct ophthalmoscope (Fig. 4B).

### Views after training

#### The fundus images presented by the Eyesi resembled the real fundus

**Fig. 5:**
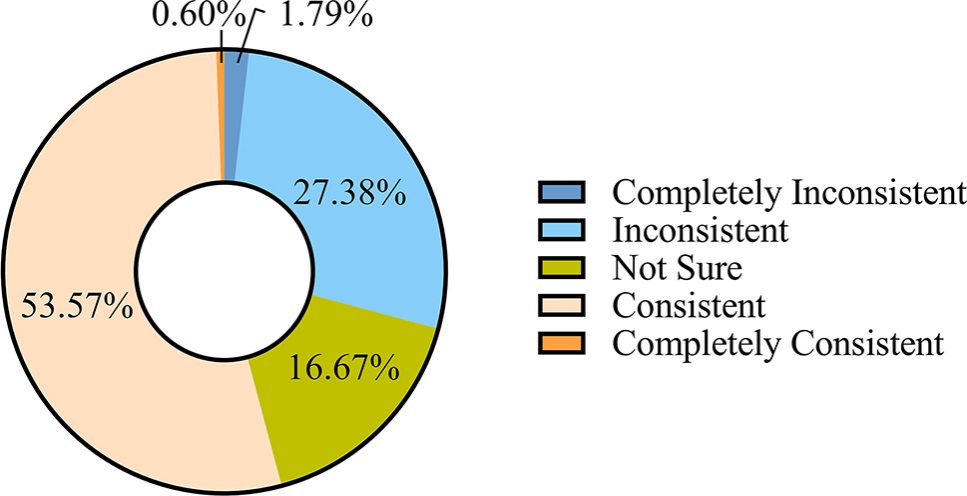
The degree of similarity between the images presented by the simulator and the real fundus according to respondents’ comments on ‘Do you think the images presented by the simulator are consistent with the real fundus?’. The questionnaire response rates for the question is 100%

After completing the training, 54.17% of the respondents believed that the images presented by the Eyesi were consistent with the real fundus, while 29.17% believed that the two images were discordant (Fig. 5).

### Kirkpatrick’s level 1 evaluation

#### The Eyesi was easier to operate and improved users’ confidence

Compared with Group B (4.71 ± 0.33), the students in Group A achieved higher scores (6.71 ± 0.21) in “the ease of use of training tool.” Furthermore, scores of “self-confidence to examine for healthy volunteers or patients with retinal diseases in future clinical practice” in Group A (7.47 ± 0.21; 6.93 ± 0.22) were higher relative to Group B (5.94 ± 0.35; 5.17 ± 0.34), *P* < 0.001 (Table 3).

**Table 3:** Difficulty and confidence in different training tools

subjective feeling	Group A M(SEM)	Group B M(SEM)	*P* value
the ease of use of training tool	6.71 (0.21)	4.71 (0.33)	< 0.001
self-confidence to examine for healthy volunteers	7.47 (0.21)	5.94 (0.35)	< 0.001
self-confidence to examine for patients with retinopathy	6.93 (0.22)	5.17 (0.34)	< 0.001

After Eyesi training, Group A; after TDO training, Group B

The values represent questionnaire statistics, with higher values indicating greater affirmation

*The Eyesi did not affect the willingness to use direct ophthalmoscope in future practice and improve interest for further learning*

**Fig. 6:**
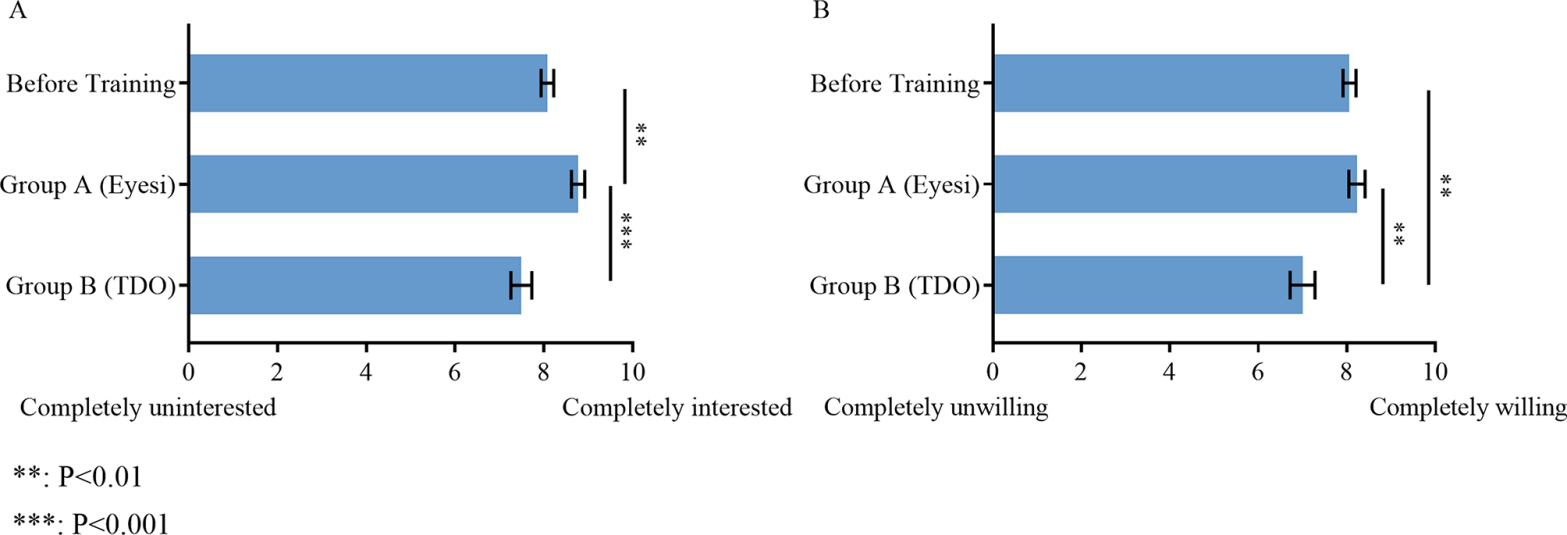
Influence of learning direct ophthalmoscope with different tools on the willingness to use the tool in the future and interest in further learning. Scores of **A** ‘Interest in further learning of direct ophthalmoscope’ and **B** ‘Willingness to use direct ophthalmoscope when fundus examination is needed in future clinical practice’ in Group A (after Eyesi training) and Group B (after TDO training) compared with that before training. The questionnaire response rates for the question represented in figure A are 85.71% before training, 96.94 in Group A and 92.86% in Group B. The questionnaire response rates for the question represented in figure B are 85.71% before training, 95.92 in Group A and 92.86% in Group B

With regard to interest for further learning, scores of Group A (8.77 ± 0.15) were significantly higher than scores obtained before training (8.08 ± 0.14, *P* < 0.01) and more than scores of Group B (7.49 ± 0.24, *P* < 0.001) (Fig. 6A). Scores of Group B about their willingness to use a direct ophthalmoscope in future clinical practice (7.00 ± 0.28) were significantly lower than that of pre-training scores (8.06 ± 0.15, *P* < 0.01) and that of Group A (8.23 ± 0.18, *P* < 0.01) (Fig. 6B).

### The vast majority of respondents suggested adding the Eyesi training

**Fig. 7:**
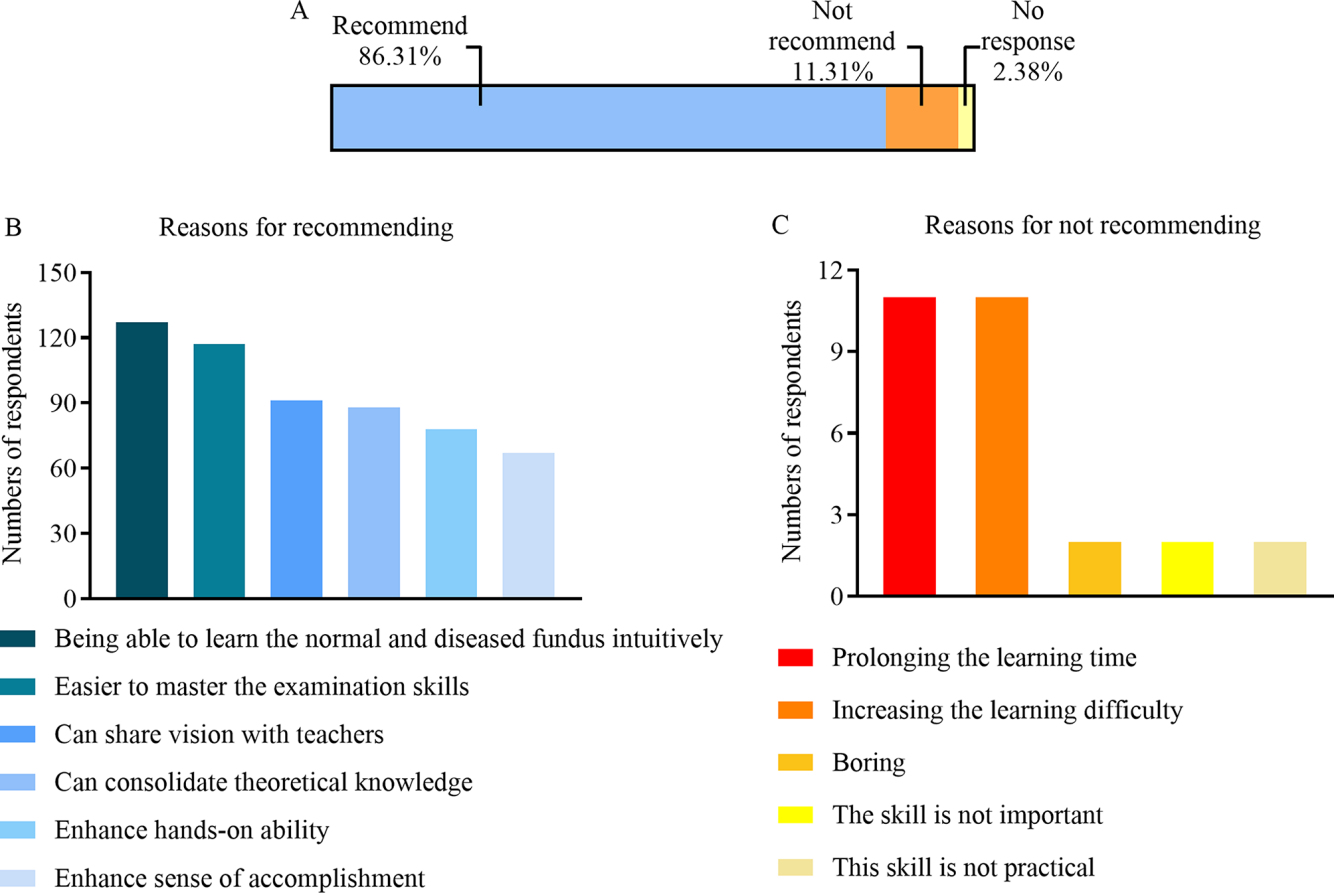
Respondents’ feedback on the inclusion of Eyesi training to ophthalmic preclinical training. **A** Percentage distribution of recommending, not recommending or expressing no difference. Reasons for **B** recommending and **C** not recommending the Eyesi direct ophthalmoscope simulator

For the item “Do you recommend adding Eyesi training to ophthalmic preclinical training?”, 145 (86.31%) respondents chose “Recommend”, while 19 (11.31%) chose “Do Not Recommend.” Among the reasons for recommending, 117 chose “being able to learn the normal and diseased fundus intuitively” and “easier to master the examination skills.” Among the reasons for not recommending, 11 chose “prolonging the learning time” and “increasing the learning difficulty” respectively (Fig. 7).

### Kirkpatrick’s level 2 evaluation

#### The Eyesi was more conducive to identifying fundus structure

In terms of the degree to which the Eyesi or TDO may help students, the average scores of the respondents in Group A were significantly higher than that of Group B (*P* < 0.001) (Table 4) with regard to: “focusing on the fundus and obtaining a clear image,” “finding the optic disc and correctly estimating the cup disc ratio,” “observing the morphology feature and distribution of retinal vessels and distinguishing between arteries and veins,” and “finding and identifying typical fundus manifestations of common retinal diseases.”

**Table 4:** Scores of identification of fundus structure

Identification of fundus structure	Group A M(SEM)	Group B M(SEM)	*P* value
focus on the fundus and obtain a clear image	7.47 (0.22)	5.50 (0.34)	< 0.001
find the optic disc and correctly estimate the cup disc ratio	7.59 (0.19)	4.09 (0.34)	< 0.001
observe the morphology feature and distribution of retinal vessels and distinguish between arteries and veins	7.51 (0.19)	4.89 (0.36)	< 0.001
find and identify typical fundus manifestations of common retinopathy	6.58 (0.24)	4.16 (0.34)	< 0.001

After Eyesi training, Group A; after TDO training, Group B

The values represent questionnaire statistics, with higher values indicating greater affirmation

#### The Eyesi training was more helpful to consolidate theoretical knowledge than traditional training

**Fig. 8:**
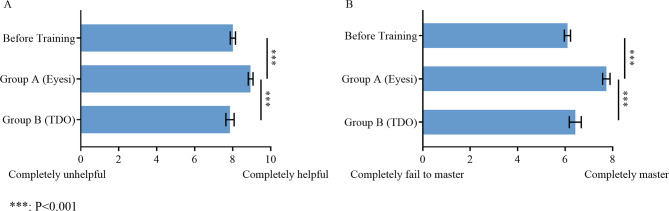
Influence of learning to use direct ophthalmoscope with different tools on mastering theoretical knowledge. Scores of **A** ‘The degree to which learning to use the direct ophthalmoscope helps consolidate relevant theoretical knowledge’ and **B** ‘Your degree of mastery of the fundus course content’ in Group A (after Eyesi training) and Group B (after TDO training) compared with that before training. The questionnaire response rates for the question represented in figure A are 85.71% before training, 96.94 in Group A and 92.86% in Group B. The questionnaire response rates for the question represented in figure B are 85.71% before training, 95.92 in Group A and 92.86% in Group B

After training, scores of Group A with regard to “the degree to which learning direct ophthalmoscope helps consolidate relevant theoretical knowledge” increased from 8.01 ± 0.14 to 8.95 ± 0.13 (*P* < 0.001). However, there was no significant change in scores of Group B (7.86 ± 0.22) (Fig. 8A). In the self-evaluation of the degree of mastering the fundus course content, scores of Group A increased from 6.10 ± 0.13 to 7.74 ± 0.16 (*P* < 0.001); similarly, there was also no significant difference in Group B (6.43 ± 0.25) (Fig. 8B).

### All participants passed the SOP test

A score of 80 or above in the sop test was considered acceptable, and all participants passed. The average score of SOP test was 93.71 ± 0.40. All operational errors were pointed out and corrected after the test of each person.

### Respondents were inclined to combine training

**Fig. 9:**
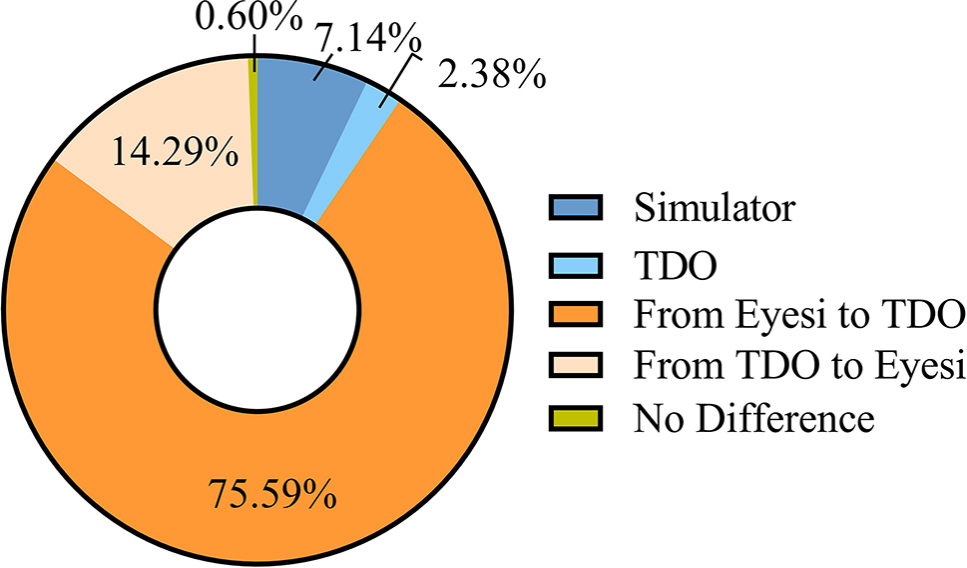
Percentage distribution of answers to the following question: Overall, which one do you prefer to learn and practice the operation of the direct ophthalmoscope? The questionnaire response rates for the question is 100%

Most participants believed that the Eyesi was more comprehensible (80.36%) and led to better learning (57.14%) and were more willing to use the tool for assessment (71.43%). However, 78.57% of respondents considered that TDO was closer to tools typically implemented in clinical practice. In terms of learning about the fundus of healthy volunteers, the proportion of the two options was similar, while 63.69% of the respondents preferred the Eyesi for learning retinal diseases (Table 5). In general, 75.59% of respondents felt inclined to practice with the Eyesi first before using TDO (Fig. 9).

**Table 5:** Respondents’ inclination between the two training tools in the specific aspect

Respondents inclination	Eyesi	TDO	No Difference
Which one is more **comprehensible**?	80.36%	11.90%	7.74%
Which one has **better learning effect**?	57.14%	31.55%	11.31%
Which one is **closer to clinical practice**?	17.26%	78.57%	4.17%
Which one do you **prefer for assessment**?	71.43%	19.64%	8.93%
Which one is better for **learning the fundus of healthy volunteers**?	44.05%	48.81%	7.14%
Which one is better for **learning the fundus of patients with retinopathy**?	63.69%	29.76%	6.55%

After all respondents completing both Eyesi and TDO training

## Discussion

Medical undergraduates often have limited specialty knowledge, clinical experience, and confidence in practice [33]. Many of them consider becoming a doctor as their long-term career plan, with some aspiring to become general practitioners [34–37]. Therefore, the primary goal of undergraduate medical education is to deepen their understanding of disease signs and symptoms and train them to skillfully perform fundamental examinations to establish valuable diagnostic foundations. Achieving this objective requires systematic and efficient guidance from teachers.

Fundus examination is vital for the diagnosis of various ophthalmological and systemic diseases. It is widely recognized that all medical students and general practitioners should possess a solid understanding and proficiency in fundus examination [38]. In our study, a vast majority of respondents agreed that direct ophthalmoscopy is commonly used, effective, and an essential component of their training. However, it was also acknowledged that mastering this technique is challenging. The Eyesi presents various fundus findings to users, enabling them to observe what a traditional ophthalmoscope would reveal. Participants expressed that Eyesi provides realistic fundus images and offers significant advantages in identifying fundus structures compared to traditional direct ophthalmoscopy. Consequently, Eyesi training proves to be feasible and meets the needs of undergraduate medical students.

We conducted a comparative analysis of operational learning and theoretical knowledge between Eyesi and TDO. Novice learners practicing with TDO may experience discomfort and increased pressure due to repeating examination steps on volunteers or patients [39]. The lack of real-time observation sharing between teachers and students hampers timely guidance, impeding students’ progress.

In contrast, the Eyesi simulator addresses these challenges. While the Eyesi simulator simulates patients’ resistance to light after prolonged examination, it remains an unrestricted practice tool for students. The Eyesi can also automatically time the illumination of the fundus and display the already examined area, reducing patient discomfort due to inexperienced operation and alleviating the psychological burden during the learning process. Moreover, the Eyesi offers the option to simulate different pupil sizes and allows for dilation, enabling students to gradually practice and repeatedly examine until they can observe comprehensive fundus structures, even under smaller pupils, thereby facilitating the learning process. Furthermore, the Eyesi assists in the theoretical learning of relevant retinal diseases. Unlike directly showing fundus images on a screen, the Eyesi simulates the real clinical scenario of using a direct ophthalmoscope, presenting fundus lesions more realistically. Additionally, it displays relevant theoretical knowledge for learning after the user marks the lesions they observe. The Eyesi compensates for the limited availability of clinical patients or cases where patients may not cooperate with students, thereby enabling students to gain insight into typical pathological conditions and reinforce relevant theoretical knowledge. These factors collectively contribute to a more engaging and effective learning experience with the Eyesi simulator. It also emphasizes the importance of performing a comprehensive fundus examination without solely relying on retinal photography, using non-examined peripheral retinal lesions as reminders for students.

The increased confidence observed in Group A can be attributed to these advantages, potentially leading to an improved utilization rate and proficiency of the direct ophthalmoscope in primary medical practice. In conclusion, this training approach aids in identifying fundus structures, enhancing students’ operational abilities, and consolidating their theoretical knowledge of the fundus. Consequently, the Eyesi simulator is expected to be a suitable choice for novices.

The majority of respondents expressed a preference for the Eyesi simulator, considering it to be easier to understand and a more effective learning tool, while TDO is actually implemented in real-world clinical practice. However, starting with TDO as the initial training method may potentially impact participants’ interest in further learning. Therefore, we recommend that undergraduates practice with the Eyesi before transitioning to the traditional method, which is consistent with the preference of most participants in this study. This sequential training method may enhance the learning experience and better prepare students for future clinical settings.

The transition from Eyesi training to using TDO in clinical applications poses multiple challenges for students. They must effectively communicate with patients during the examination process, master precise positioning techniques due to potential instability in patients’ eyes, and adapt to varying pupil sizes. Additionally, the simulator’s limitation to simulating typical retinal lesions contrasts with the diverse and rare conditions encountered in actual clinical practice, necessitating comprehensive understanding and recognition abilities. Furthermore, this transition may lead to confidence and anxiety issues among students. It is important to emphasize that the Eyesi serves as a complement to, rather than a replacement for, real patient experiences in the clinical setting. To address these challenges, educators can offer practical opportunities through simulated clinical practices and real patient training, encouraging students to participate in clinical internships to enhance their clinical competence.

To further investigate whether the Eyesi is a superior training tool, we compared our study with previous studies. With the advancement of technology and the application of virtual reality (VR) in medical education, we have gradually phased out the use of slides or photographs to simulate the fundus in simulators, opting instead for designs that are more closely aligned with clinical practice [40, 41]. In terms of its design advantages, the Eyesi distinguishes itself by closely simulating the shape and function of a real direct ophthalmoscope, utilizing a handheld device and a head-face model, making it more akin to the tools used in actual clinical practice compared to other devices [42–44]. Unlike other simulators utilizing VR technology, the Eyesi does not require wearing bulky VR goggles, and according to our questionnaire results, it more accurately simulates the real fundus state [45]. Additionally, the Eyesi’s feature of enabling teachers to provide real-time guidance through a monitoring screen is a valuable asset. Furthermore, its built-in case database of retinal diseases allows students to learn independently and addresses the limitations of TDO and other simulators [41]. In comparison to other study utilizing the Eyesi, and as opposed to conducting separate studies sequentially, our research adopted a parallel-group design to more comprehensively assess participants’ perceptions of different tools simultaneously [46]. Another study focusing on the Eyesi evaluated only the impact of different tools on students’ confidence but also concluded it to be a beneficial teaching tool [47]. Regarding the Eyesi’s indirect ophthalmoscope simulator, existing researches have compared the examination time and detection scores of physicians using this tool, which were superior to those of medical students, further supporting the effectiveness of incorporating the Eyesi simulator into training as a supplementary teaching tool [48, 49]. The number of respondents in our study was significantly larger than that of past studies [25, 50].

From the collected opinions, we found that participants appreciated the Eyesi’s user-friendliness, clarity, adjustable pupil size, and the presentation of typical cases. However, the limited time for practice was noted as an area of improvement. And some challenges with TDO were highlighted, such as a small vision field hindering the identification of common fundus lesions and greater difficulty in operation. Nevertheless, students appreciated its realism and the ability to receive feedback from volunteers. Despite the favorable feedback for the Eyesi, a few students did not fully support its use. To address these concerns, we plan to enhance future courses by allocating additional practice time and thoughtfully selecting built-in cases within the Eyesi.

As for study design, we designed four questionnaires to evaluate the subjective feelings of respondents before and after training and on different training tools, as well as their confidence and willingness to use a direct ophthalmoscope in future work. We also compared undergraduates’ tendencies for different learning purposes, and offered suggestions on learning sequences. Self-reporting questionnaires provide valuable insights into participants’ subjective perceptions, feedback, and satisfaction with course content and training methods [51]. These assessments shed light on the training’s potential benefits in enhancing learners’ skills and self-assurance, allowing us to optimize the program accordingly for a more engaging learning experience. In our study, we have implemented multiple strategies to mitigate potential biases. Firstly, random group assignment was employed to ensure balanced representation in each training group, minimizing selection bias. Secondly, we crafted unbiased survey questions to prevent information bias. Moreover, measures such as respondent anonymity, blind data entry, and analysis were implemented to mitigate response and observer bias during data processing. While we acknowledge the limitations of self-reporting, we have taken meticulous steps to enhance the validity and reliability of our findings within the scope of our study’s constraints.

However, it is essential to establish more objective evaluation indicators, such as written exams and scenario simulation assessments, to effectively measure the enhancement of participants’ theoretical knowledge and practical skills resulting from the utilization of various training methodologies. Additionally, for evaluating the effectiveness of the Kirkpatrick model’s third and fourth levels, long-term observations will be required to assess the participants’ skills and performance in their daily clinical practices. While we acknowledge the current limitations, it is also essential to conduct further research by integrating feedback from both supervising physicians and patients to achieve a comprehensive understanding of the training outcomes in clinical settings. Recognizing this, we are planning a follow-up study that will address these concerns by focusing on specialized physicians who have undergone the training and gathering insights from experienced providers or faculty regarding the Eyesi. This subsequent research will encompass a more comprehensive assessment, including evaluation by teachers in clinical practice and a blinded evaluation methodology. Furthermore, before commencing further research, we will validate the psychometrics of our questionnaires and further refine them to better meet our survey needs.

In general, the Eyesi has shown potential advantages over the traditional direct ophthalmoscope in fundus observation, operational practice, and theoretical learning. Its user-friendly interface and intuitive design indicate it might effectively assist medical students acquiring fundus examination skills during clinical training. The positive reception of the Eyesi by most undergraduates suggests it could be a valuable contribution to ophthalmic preclinical training. This may help promote the use of the direct ophthalmoscope in primary medical institutions and, as a result, contribute to facilitating the early screening and diagnosis of retinal diseases, and aid in evaluating retinal microvascular abnormalities among patients with systemic diseases. This, in turn, has the potential to reduce healthcare costs and preserve medical resources.

### Electronic supplementary material

Below is the link to the electronic supplementary material.

Supplementary Material 1

Supplementary Material 2

Supplementary Material 3

## Data Availability

The datasets used and analysed during the current study available from the corresponding author on reasonable request.

## Declarations

### Declarations of scientific writing assistance

We thank Editage (www.editage.cn) for English language editing.

### Declarations of Generative AI and AI-assisted technologies in the writing process assistance

During the preparation of this work the authors used ChatGPT to refine the language and enhance readability. After using this tool, the authors reviewed and edited the content as needed and take full responsibility for the content of the publication.

### Practice points

- The Eyesi is superior to TDO in fundus observation, operational practice, and theoretical learning.
- We designed four questionnaires to evaluate the subjective feelings of respondents comprehensively.
- We offered suggestions on learning sequence of the Eyesi and TDO.
- The Eyesi may promote the use of TDO in primary medical institutions.
