# Supplementary material for: Eyesi direct ophthalmoscope simulator: an effective training tool for medical undergraduates
Source: BMC Med Educ. 2024 Jul 20;24:783. doi: 10.1186/s12909-024-05780-w (PMC11265108; doi:10.1186/s12909-024-05780-w)
Supplement: Supplementary file 1 — Supplementary Material 1 [file 12909_2024_5780_MOESM2_ESM.docx]

Supplementary Table 6

**Score sheet for standard operation procedure of direct ophthalmoscope**

NAME:

| Scoring Item | Points | Standard Score | | Final Score |
| --- | --- | --- | --- | --- |
| Preparation | Explain fundus examination to the patient. | 2 | 10 |  |
|  | Adjust the light intensity of examination room. | 2 |  |  |
|  | Adjust the height of seats so that the patient’s head is slight below the examiner’s eyes. | 2 |  |  |
|  | Check the instrument. Clean hands and wear a mask. | 2 |  |  |
|  | Ask patients to remove his glasses and watch a distant sign. | 2 |  |  |
| Steps | Examine patient’s right eye first with examiner’s right hand holding the instrument and stand in the same side of examined eye. | 10 | 30 |  |
|  | Adjust direct ophthalmoscope to a large aperture and a convex lens of +8 to +10D, and observe the dioptric medium. | 10 |  |  |
|  | Stop down the aperture, adjust diopter, get closer to patient and focus on the retina. | 10 |  |  |
|  | Observe optic disc and report its shape, boundary, color and C/D ratio. | 15 | 40 |  |
|  | Observe peripheral retina along blood vessels. | 15 |  |  |
|  | Ask the patient to look at the light source and examine macula lutea. | 10 |  |  |
|  | Turn off the power and turn diopter to zero. | 10 | 20 |  |
|  | Record inspection results correctly. | 10 |  |  |
| Total | | 100 | |  |
