# Supplementary material for: Eyesi direct ophthalmoscope simulator: an effective training tool for medical undergraduates
Source: BMC Med Educ. 2024 Jul 20;24:783. doi: 10.1186/s12909-024-05780-w (PMC11265108; doi:10.1186/s12909-024-05780-w)
Supplement: Supplementary file 2 — Supplementary Material 2 [file 12909_2024_5780_MOESM3_ESM.docx]

Supplementary Table 1

**Teaching investigation of direct ophthalmoscopy simulator in ophthalmic preclinical training (pre-training questionnaire)**

Hello everyone! Welcome to ophthalmic preclinical training. In order to understand the necessity of adding direct ophthalmoscope simulator among undergraduates and arrange the teaching more reasonably, we carried out this questionnaire survey. **The questionnaire information will be kept strictly confidential and only used for this teaching survey. Please fill in truthfully, thank you!**

**1. Your Age：**_________________

**2. Your Gender：** ○A. male ○B. Female

**3. Have you completed the theoretical course of Ophthalmology？** ○A. Yes ○B. No

**4. Department that you has completed before ophthalmic preclinical training:** (multiple choice)

□A. Internal Medicine Department

□B. Surgery Department

□C. Obstetrics and Gynecology Department □D. Pediatric Department

□E. Dermatology Department

□F. E.N.T. Department

□G. Neurology Department

□H. Emergency Department

□I. Department of Infectious Diseases

□J. Oncology Department

□K. Psychiatry Department

□L. None

**5. Have you attended any ophthalmic training beforehand this training？**

○A. Yes；Time（year/month）： ○B. No

**6. Have you received systematic direct ophthalmoscopy training?**

○A. Yes；Time（year/month）： ○B. No

**7. For the following items, please select the one that best matches your subjective perception (represented by ‘√’)**

|  | **Completely Agree** | **Agree** | **Not Sure** | **Disagree** | **Completely Disagree** |
| --- | --- | --- | --- | --- | --- |
| **1.** **Direct ophthalmoscope is one of the most commonly used inspection tools in clinical ophthalmology.** |  |  |  |  |  |
| **2. Direct ophthalmoscope is** **an effective inspection method for the diagnosis of retinal diseases.** |  |  |  |  |  |
| **3. Learning to use the direct ophthalmoscope is difficult.** |  |  |  |  |  |
| **4. The examination steps involved in using the direct ophthalmoscope are complex.** |  |  |  |  |  |

**For the following questions, please select the most appropriate number according to your subjective perception, represented by ‘√’.**

**8. Your mastery degree of fundus course content:**

Completely fail to master 1 2 3 4 5 6 7 8 9 10 Completely master

**9. Are you interested in learning to operate the direct ophthalmoscope?**

Completely uninterested 1 2 3 4 5 6 7 8 9 10 Completely interested

**10. The degree to which learning direct ophthalmoscope helps consolidate relevant theoretical knowledge.**

Completely unhelpful 1 2 3 4 5 6 7 8 9 10 Completely helpful

**11. Do you think it is necessary for non-ophthalmologists to master the use of the direct ophthalmoscope?**

Completely unnecessary 1 2 3 4 5 6 7 8 9 10 Completely necessary

**12. Willingness to use direct ophthalmoscope when fundus examination is needed in future clinical practice.**

Completely unwilling 1 2 3 4 5 6 7 8 9 10 Completely willing

| Dimension | Question |
| --- | --- |
| the importance of learning direct ophthalmoscope operation | 7.1, 7.2, 11 |
| competency in direct ophthalmoscope operation | 7.3, 7.4 |
| level of theoretical knowledge related to direct ophthalmoscope | 8, 10 |
| interest in further learning | 9, 12 |

Supplementary Table 2

**Teaching investigation of direct ophthalmoscopy simulator in** **ophthalmic preclinical training (after Eyesi direct ophthalmoscope simulator training)**

The questionnaire information will be kept strictly confidential and only used for this teaching survey. Please fill in truthfully. Thank you for your time and effort to improve the quality of ophthalmic preclinical training!

1. **Which learning tool did you use?**

A. Eyesi direct ophthalmoscope simulator B. Traditional direct ophthalmoscope

1. **Your actual practice time was about:**

○A. less than 15min ○B. 15min-30min

○C. 30min-45min ○D. 45min-60min ○E. more than 60min

1. **Was your practicing done under normal pupil or dilated pupil?**

○A. normal pupil ○B. dilated pupil ○C. both

1. **Was the teacher's guidance helpful in the learning process?**

Completely unhelpful 1 2 3 4 5 6 7 8 9 10 Completely helpful

1. **Did you have enough time to practice during the learning process？**

Completely inadequate 1 2 3 4 5 6 7 8 9 10 Completely adequate

1. **What do you think are the important steps in practicing direct ophthalmoscopy?**

(single or multiple options)

○A. Explanation, demonstration and operation guidance

○B. Get a sense of accomplishment from the operation

○C. Normal fundus images can be observed successfully

○D. Fundus images of typical diseases can be observed successfully

○E. Plenty of time to practice

○F. Else, please write down

1. **After training, how confident you are in your operation with direct ophthalmoscope?**

Completely unconfident 1 2 3 4 5 6 7 8 9 10 Completely confident

1. **The ease of use of training tool:**

Very hard 1 2 3 4 5 6 7 8 9 10 Very easy

1. **Ease of use:**
2. focus on the fundus and obtain a clear image:

Very hard 1 2 3 4 5 6 7 8 9 10 Very easy

1. find the optic disc and correctly estimate the cup disc ratio:

Very hard 1 2 3 4 5 6 7 8 9 10 Very easy

1. observe the morphology feature and distribution of retinal vessels and distinguish between arteries and veins:

Very hard 1 2 3 4 5 6 7 8 9 10 Very easy

1. find and identify typical fundus manifestations of common retinal diseases:

Very hard 1 2 3 4 5 6 7 8 9 10 Very easy

1. **The degree to which learning to use direct ophthalmoscope helps consolidate relevant theoretical knowledge.**

Completely unhelpful 1 2 3 4 5 6 7 8 9 10 Completely helpful

1. **After training, your degree of mastery of the fundus course content:**

Completely fail to master 1 2 3 4 5 6 7 8 9 10 Completely master

1. **The need for non-ophthalmologists to master the operation of direct ophthalmoscope:**

Completely unnecessary 1 2 3 4 5 6 7 8 9 10 Completely necessary

1. **Interest in further learning of direct ophthalmoscope:**

Completely uninterested 1 2 3 4 5 6 7 8 9 10 Completely interested

1. **Your self-confidence to use direct ophthalmoscopy to examine for healthy volunteers in clinical practice:**

Completely unconfident 1 2 3 4 5 6 7 8 9 10 Completely confident

1. **Your self-confidence to use direct ophthalmoscopy to examine for patients with retinal diseases in clinical practice:**

Completely unconfident 1 2 3 4 5 6 7 8 9 10 Completely confident

1. **Your willingness to use direct ophthalmoscope when fundus examination is needed in future clinical practice**

Completely unwilling 1 2 3 4 5 6 7 8 9 10 Completely willing

1. **Whether it is necessary to increase the practice time? (- means to reduce the practice time,+ means to increase)**

A．-1h B.-0.5h C.0 D.+0.5h E.+1h F. The practice time is not enough at all.

1. **Do you have any other difficulty and doubts about direct ophthalmoscopy?**

| Dimension | Question |
| --- | --- |
| the importance of learning direct ophthalmoscope operation | 12 |
| competency in direct ophthalmoscope operation | 7, 8, 9.1-9.4, 14, 15 |
| level of theoretical knowledge related to direct ophthalmoscope | 10, 11 |
| interest in further learning | 13, 16 |

Supplementary Table 3

**Teaching investigation of direct ophthalmoscopy simulator in ophthalmic preclinical training (after traditional direct ophthalmoscope training)**

The questionnaire information will be kept strictly confidential and only used for this teaching survey. Please fill in truthfully. Thank you for your time and effort to improve the quality of ophthalmic preclinical training!

1. **Which learning tool did you use?**

A. Eyesi direct ophthalmoscope simulator B. Traditional direct ophthalmoscope

1. **Your actual practice time was about:**

○A. less than 15min ○B. 15min-30min

○C. 30min-45min ○D. 45min-60min ○E. more than 60min

1. **Was your practicing done under normal pupil or dilated pupil?**

○A. normal pupil ○B. dilated pupil ○C. both

1. **Was the teacher's guidance helpful in the learning process?**

Completely unhelpful 1 2 3 4 5 6 7 8 9 10 Completely helpful

1. **Did you have enough time to practice during the learning process？**

Completely inadequate 1 2 3 4 5 6 7 8 9 10 Completely adequate

1. **What do you think are the important steps in practicing direct ophthalmoscopy?**

(single or multiple options)

○A. Explanation, demonstration and operation guidance

○B. Get a sense of accomplishment from the operation

○C. Normal fundus images can be observed successfully

○D. Fundus images of typical diseases can be observed successfully

○E. Plenty of time to practice

○F. Else, please write down

1. **After training, how confident you are in your operation with direct ophthalmoscope?**

Completely unconfident 1 2 3 4 5 6 7 8 9 10 Completely confident

1. **The ease of use of training tool:**

Very hard 1 2 3 4 5 6 7 8 9 10 Very easy

1. **Ease of use:**
2. focus on the fundus and obtain a clear image:

Very hard 1 2 3 4 5 6 7 8 9 10 Very easy

1. find the optic disc and correctly estimate the cup disc ratio:

Very hard 1 2 3 4 5 6 7 8 9 10 Very easy

1. observe the morphology feature and distribution of retinal vessels and distinguish between arteries and veins:

Very hard 1 2 3 4 5 6 7 8 9 10 Very easy

1. find and identify typical fundus manifestations of common retinal diseases:

Very hard 1 2 3 4 5 6 7 8 9 10 Very easy

1. **The degree to which learning to use direct ophthalmoscope helps consolidate relevant theoretical knowledge.**

Completely unhelpful 1 2 3 4 5 6 7 8 9 10 Completely helpful

1. **After training, your degree of mastery of the fundus course content:**

Completely fail to master 1 2 3 4 5 6 7 8 9 10 Completely master

1. **The need for non-ophthalmologists to master the operation of direct ophthalmoscope:**

Completely unnecessary 1 2 3 4 5 6 7 8 9 10 Completely necessary

1. **Interest in further learning of direct ophthalmoscope:**

Completely uninterested 1 2 3 4 5 6 7 8 9 10 Completely interested

1. **Your self-confidence to use direct ophthalmoscopy to examine for healthy volunteers in clinical practice:**

Completely unconfident 1 2 3 4 5 6 7 8 9 10 Completely confident

1. **Your self-confidence to use direct ophthalmoscopy to examine for patients with retinal diseases in clinical practice:**

Completely unconfident 1 2 3 4 5 6 7 8 9 10 Completely confident

1. **Your willingness to use direct ophthalmoscope when fundus examination is needed in future clinical practice**

Completely unwilling 1 2 3 4 5 6 7 8 9 10 Completely willing

1. **Whether it is necessary to increase the practice time? (- means to reduce the practice time,+ means to increase)**

A．-1h B.-0.5h C.0 D.+0.5h E.+1h F. The practice time is not enough at all.

1. **Do you have any other difficulty and doubts about direct ophthalmoscopy?**

| Dimension | Question |
| --- | --- |
| the importance of learning direct ophthalmoscope operation | 12 |
| competency in direct ophthalmoscope operation | 7, 8, 9.1-9.4, 14, 15 |
| level of theoretical knowledge related to direct ophthalmoscope | 10, 11 |
| interest in further learning | 13, 16 |

Supplementary Table 4

**Teaching investigation of direct ophthalmoscopy simulator in ophthalmic preclinical training (summary questionnaire )**

The questionnaire information will be kept strictly confidential and only used for this teaching survey. Please fill in truthfully. Thank you for your time and effort to improve the quality of ophthalmic preclinical training!

1. **Do you think the images presented by the simulator are consistent with the real fundus?**

○A .Completely Inconsistent ○B. Inconsistent ○C. Not Sure

○D. Consistent ○E. Completely Consistent

1. **Which training tool is more comprehensible?**

○A. Eyesi direct ophthalmoscope simulator ○B. Traditional direct ophthalmoscope ○C. No difference

1. **Which training tool has better learning effect?**

○A. Eyesi direct ophthalmoscope simulator ○B. Traditional direct ophthalmoscope ○C. No difference

1. **Which training tool is closer to clinical practice?**

○A. Eyesi direct ophthalmoscope simulator ○B. Traditional direct ophthalmoscope ○C. No difference

1. **Which training tool is better for learning the fundus of healthy volunteers?**

○A. Eyesi direct ophthalmoscope simulator ○B. Traditional direct ophthalmoscope ○C. No difference

1. **Which training tool is better for learning the fundus of patients with retinal diseases?**

○A. Eyesi direct ophthalmoscope simulator ○B. Traditional direct ophthalmoscope ○C. No difference

1. **Which training tool do you prefer for assessment?**

○A. Eyesi direct ophthalmoscope simulator ○B. Traditional direct ophthalmoscope ○C. No difference

1. **Overall, which training tool do you prefer to learn and practice the operation of the direct ophthalmoscope?**

○A. Eyesi direct ophthalmoscope simulator

○B. Traditional direct ophthalmoscope

○C. Combined, from A to B

○D. Combined, from B to A

○E. No difference

Your reasons:

1. **Do you recommend adding simulator training to ophthalmic preclinical training?**

**○****Recommend, because（multiple choice）：**

A. Easier to master the examination skills; B. Can share vision with teachers;

C. Being able to learn the normal and diseased fundus intuitively;

D. Can consolidate theoretical knowledge; E. Enhance hands-on ability; F. Enhance sense of accomplishment;

G. others

**○Not Recommend, because（multiple choice）：**

A. Prolonging the learning time; B. Increasing the learning difficulty;

C. It is boring; D. The skill is not important; E. The skill is not practical;

F. Others
