# Supplementary material for: Eyesi direct ophthalmoscope simulator: an effective training tool for medical undergraduates
Source: BMC Med Educ. 2024 Jul 20;24:783. doi: 10.1186/s12909-024-05780-w (PMC11265108; doi:10.1186/s12909-024-05780-w)
Supplement: Supplementary file 3 — Supplementary Material 3 [file 12909_2024_5780_MOESM1_ESM.docx]

Supplementary Table 5: The Cronbach's alpha and composite reliability statistics of four dimensions.

|  |  | **Before training** | | | **After training** | | |
| --- | --- | --- | --- | --- | --- | --- | --- |
|  | | Cronbach's alpha | Composite reliability | Items number | Cronbach's alpha | Composite reliability | Items number |
| **Importance** | | 0.528 | 0.601 | 3 | - | - | 1 |
| **Operation** | | 0.748 | 0.816 | 2 | 0.93 | 0.931 | 8 |
| **Theory** | | 0.534 | 0.637 | 2 | 0.741 | 0.741 | 2 |
| **Interest** | | 0.641 | 0.674 | 2 | 0.84 | 0.839 | 2 |
